# Assessing the Role of CD103 in Immunity to an Intestinal Helminth Parasite

**DOI:** 10.1371/journal.pone.0019580

**Published:** 2011-05-05

**Authors:** Sarah C. Mullaly, Kyle Burrows, Frann Antignano, Colby Zaph

**Affiliations:** 1 The Biomedical Research Centre, University of British Columbia, Vancouver, British Columbia, Canada; 2 Department of Pathology and Laboratory Medicine, University of British Columbia, Vancouver, British Columbia, Canada; McGill University, Canada

## Abstract

**Background:**

In the intestine, the integrin CD103 is expressed on a subset of T regulatory (T_reg_) cells and a population of dendritic cells (DCs) that produce retinoic acid and promote immune homeostasis. However, the role of CD103 during intestinal helminth infection has not been tested.

**Methodology/Principal Findings:**

We demonstrate that CD103 is dispensable for the development of protective immunity to the helminth parasite *Trichuris muris*. While we observed an increase in the frequency of CD103^+^ DCs in the lamina propria (LP) following acute high-dose infection with *Trichuris*, lack of CD103 had no effect on the frequency of CD11c^+^ DCs in the LP or mesenteric lymph nodes (mLN). CD103-deficient (CD103^−/−^) mice develop a slightly increased and earlier T cell response but resolve infection with similar kinetics to control mice. Similarly, low-dose chronic infection of CD103^−/−^ mice with *Trichuris* resulted in no significant difference in immunity or parasite burden. Absence of CD103 also had no effect on the frequency of CD4^+^CD25^+^Foxp3^+^ T_reg_ cells in the mLN or LP.

**Conclusions/Significance:**

These results suggest that CD103 is dispensable for intestinal immunity during helminth infection. Furthermore, lack of CD103 had no effect on DC or T_reg_ recruitment or retention within the large intestine.

## Introduction

Mucosal sites such as the gastrointestinal and respiratory tracts are primary entry points for both innocuous and pathogenic agents, and multiple mechanisms control the balance between the development of immunity, regulation of inflammation and induction of tolerance [1]. A better understanding of the mechanisms underlying intestinal homeostasis is key to the development of new therapeutics for various inflammatory conditions, including inflammatory bowel disease. Within the gut-associated lymphoid tissues of the intestine, CD4^+^CD25^+^Foxp3^+^ regulatory T (T_reg_) cells and dendritic cells (DCs) are critical in the maintenance of mucosal immune homeostasis and subsets of these T_reg_ cells and DCs express the E-cadherin receptor, αE(CD103)β7 integrin [2], [3], [4], [5], [6]. Thus it has been suggested that CD103 is a critical component of intestinal immune homeostasis.

CD103-deficient mice were initially shown to have reduced numbers of intestinal intraepithelial lymphocytes and lamina propria (LP) T cells, suggesting a role for this integrin in the localization of T cells within intestinal tissue [7]. Indeed, CD103 plays a critical role in site-specific T_reg_ cell retention in the skin under both resting conditions and following *Leishmania major* infection [8]. However, CD103 is not required for T_reg_ cell function in the intestine in a T cell transfer model of colitis, although protection from inflammation is contingent upon non-T cell expression of CD103 [9].

CD103^+^ DCs represent ∼10–50% of mesenteric lymph node (mLN) DCs [5], [9], [10] and are specialized to generate CCR9^+^ α4β7 integrin^+^ small intestine-tropic T cells [11], [12], [13] and Foxp3^+^ T_reg_ cells [14] primarily through the production of retinoic acid [10], [14], [15], [16], [17], [18]. In addition, LP CD103^+^ DCs are critical for the transport of *Salmonella enterica* (subspecies 1 serovar Typhimurium) from the intestine to the mLN following oral infection [5], indicating that CD103 plays an important role in intestinal immunosurveillance. Despite the broad spectrum of cellular functions for CD103, its role in large intestinal immunity following parasite infection has not been examined.

*Trichuris muris* is a helminth parasite of mice that provides a powerful model to analyze the cellular and molecular factors required for the development of resistance and susceptibility to infection. For example, acute infection of C57BL/6 mice with a high dose (200 eggs) of *Trichuris* results in a polarized Th2 cell response, characterized by high levels of IL-4, IL-5 and IL-13, and resistance to infection. By contrast, chronic infection of C57BL/6 mice with a low dose (30 eggs) of *Trichuris* leads to a Th1 cell response, production of IFN-γ and failure to clear parasites [19]. Employing this infection model, we sought to examine the role of CD103 during helminth infection.

Our results demonstrate that CD103 is not required for the development of immune responses in the intestine. While CD103-deficient (CD103^−/−^) mice developed a slightly exaggerated immune response following helminth infection, parasite expulsion was unaffected. We also observed no differences in the frequencies of T_reg_ cells or DCs in the absence of CD103, suggesting that CD103 is not required for T_reg_ cell or DC recruitment or retention in the intestine. Taken together, these results show that CD103 is not absolutely required for the development of intestinal immunity during helminth infection.

## Results

### CD103-deficient mice are resistant to acute *Trichuris* infection

**Figure 1:**
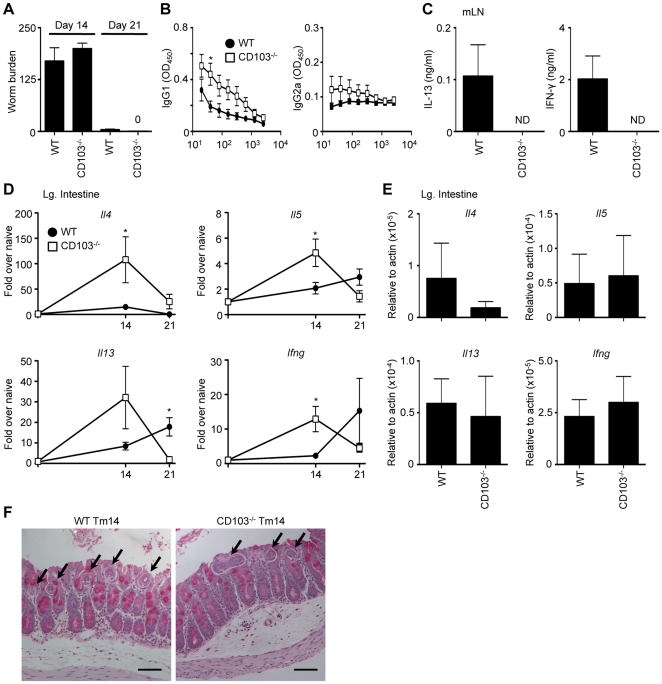
CD103-deficient mice are resistant to acute *Trichuris* infection. WT and CD103^−/−^ mice were orally infected with 200 *Trichuris* eggs. (**A**) Number of worms per mouse was determined microscopically at days 14 and 21 following infection. (**B**) *Trichuris*-specific IgG1 and IgG2a levels were assessed by ELISA from the sera of 21-day infected WT (•) and CD103^−/−^ (□) mice. (**C**) mLN cells from WT and CD103^−/−^ mice were restimulated with anti-CD3/CD28 Abs for 72 h and supernatants were analyzed by ELISA for production of IL-13 and IFN-γ. (**D–E**) Expression of *Il4*, *Il5*, *Il13* and *Ifng* mRNA levels in the large intestine were assessed by qPCR (**D**) at days 14 and 21 following infection and (**E**) in naïve animals. Data are expressed as relative to uninfected control mice (**D**) and relative to actin (**E**). (**F**) Representative images of PAS-stained cecal morphology from 14-day *Trichuris*-infected (Tm14) WT and CD103^−/−^ mice. Arrows indicate worms. Images were captured at 100× magnification and scale bar represents 100 µm. (**A**,**D**,**E**) Data are averaged from 2–3 experiments (n = 6–12); (**B**,**C**) Data is representative of 2–3 experiments (n = 8–12). ND = not detected.

Following infection with 200 *Trichuris* eggs (high dose), wild-type C57BL/6 (WT) mice develop a polarized CD4^+^ Th2 cell response characterized by high levels of IL-4, IL-5 and IL-13 [20]. To directly test whether CD103 was required for the development of protective immunity, WT and CD103^−/−^ mice were acutely infected with a high dose of *Trichuris* eggs. Similar to WT mice, CD103^−/−^ mice were resistant to *Trichuris*, expelling worms by day 21 after equivalent establishment of infection at day 14 (**Figure 1A**). *Trichuris*-specific serum IgG1 titers, a hallmark of systemic Th2 cell responses, were increased in CD103^−/−^ mice compared to infected WT mice, while IFN-γ-dependent IgG2a titers were similar between WT and CD103^−/−^ mice (**Figure 1B**). While WT mice produced measurable levels of both IL-13 and IFN-γ following restimulation of mLN cells from 21-day infected mice, we were unable to detect cytokine secretion from CD103^−/−^ mice (**Figure 1C**). Taken together with the results showing that CD103^−/−^ mice cleared infection, we hypothesized that the lack of CD103 resulted in an earlier and exacerbated Th2 cell response. Consistent with this, intestinal gene expression levels of *Il4*, *Il5* and *Il13* at day 14 post-infection were heightened in CD103^−/−^ mice (**Figure 1D**). However, we also observed significantly increased induction of *Ifng* in CD103^−/−^ mice at day 14, suggesting that the absence of CD103 may result in a global dysregulation of T cell responses. Under naïve conditions, *Il4*, *Il5*, *Il13* and *Ifng* expression levels were not significantly different in CD103^−/−^ mice when compared to WT mice (**Figure 1E**). At day 14 post-infection, we observed no differences in intestinal architecture, goblet cell hyperplasia or worm burden between WT and CD103^−/−^ mice (**Figure 1F**). Thus, following acute infection with *Trichuris*, CD103^−/−^ mice develop an earlier and exacerbated immune response and are resistant to infection.

### Chronic *Trichuris* infection in CD103-deficient mice

**Figure 2:**
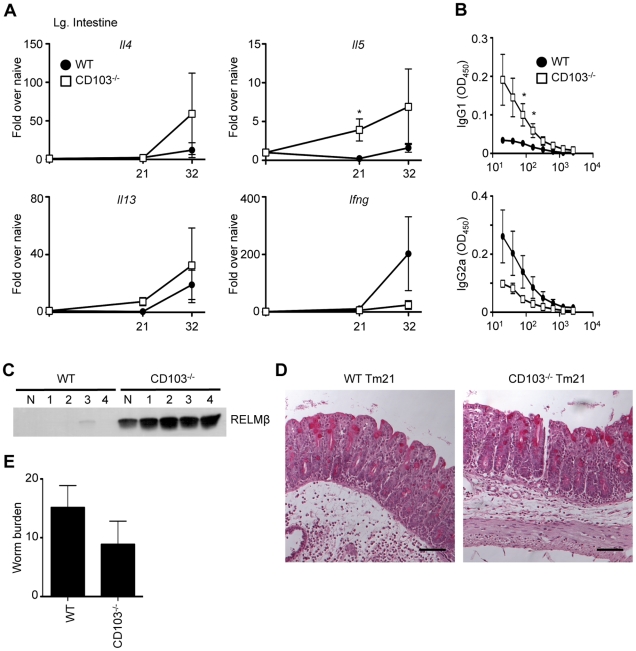
Chronic *Trichuris* infection in CD103-deficient mice. WT and CD103^−/−^ mice were orally infected with 25–30 *Trichuris* eggs. (**A**) Expression of *Il4*, *Il5*, *Il13* and *Ifng* mRNA levels in the large intestine were assessed by qPCR at days 21 and 32. (**B**) *Trichuris*-specific IgG1 and IgG2a levels were assessed by ELISA from the sera of 21-day infected WT (•) and CD103^−/−^ (□) mice. (**C**) Fecal samples from one uninfected (N) and four *Trichuris*-infected (1–4) WT and CD103^−/−^ mice were collected on day 21 and RELMβ protein levels were assessed by Western blot. (**D**) Representative images of PAS-stained cecal morphology from 21-day *Trichuris*-infected (Tm21) WT and CD103^−/−^ mice. Images were captured at 100× magnification and scale bar represents 100 µm. (**E**) Worm expulsion was assessed at day 32 following infection. (**A**,**D**) Data are averaged from 2 independent experiments (n = 6–8); (**B**,**C**) Data are representative of 2 independent experiments (n = 7–8).

A powerful aspect of the *Trichuris* model is the ability to affect the polarization of the host immune response simply by manipulating the administered dose of *Trichuris* eggs. While WT mice generate a protective Th2-biased immune response to a high dose of *Trichuris*, a low dose (30 eggs) of *Trichuris* induces a Th1 cell response resulting in chronic infection [21]. As CD103^−/−^ mice developed heightened T cell-dependent immune responses following acute infection, we hypothesized that a chronic, low dose *Trichuris* infection would also result in heightened T cell responses. However, low-dose infected CD103^−/−^ mice had higher fold induction of *Il4*, *Il5* and *Il13* and lower induction of *Ifng* mRNA in the intestine (**Figure 2A**). Consistent with the increased induction of Th2 cytokine genes, infected CD103^−/−^ mice also had elevated *Trichuris*-specific serum IgG1 and reduced serum IgG2a titers compared with infected WT mice (**Figure 2B**). Expression of resistin-like molecule β (RELMβ), a goblet cell specific protein associated with Th2 cell responses and expulsion of *Trichuris* [22], was detectable only in CD103^−/−^ mice (**Figure 2C**). However, intestinal tissue of infected WT and CD103^−/−^ mice displayed no significant differences histologically (**Figure 2D**). Furthermore, CD103^−/−^ mice failed to eradicate parasites (**Figure 2E**), suggesting that the magnitude of the increased Th2 cell response was insufficient to induce immunity to infection. Nevertheless, CD103 deficiency appears to result in a specific enhancement of Th2 responses following chronic intestinal helminth infection, rather than a global dysregulation of mucosal immunity.

### CD103 is dispensable for DC recruitment and retention in the intestine following *Trichuris* infection

**Figure 3:**
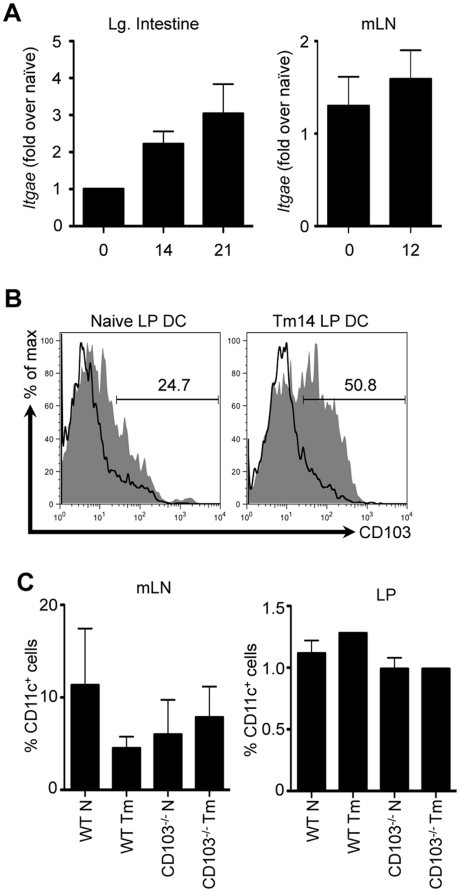
DC recruitment and retention in the intestine following *Trichuris* infection. WT and CD103^−/−^ mice were orally infected with 200 *Trichuris* eggs. (**A**) *Itgae* mRNA expression was assessed in the large intestine (left panel) and mLN (right panel) of WT mice by qPCR at days 14 and 21 following infection. (**B**) Representative data of CD103 expression on CD11c^+^ LP DCs was assessed by flow cytometry at day 14 following infection; shaded histogram: WT mice, open histogram: CD103^−/−^ mice. (**C**) Frequency of CD11c^+^ cells in the mLN (left panel) and LP (right panel) of naïve and 14-day infected WT and CD103^−/−^ mice. (**A, and left panel of C**) Data are averaged from 2–4 experiments (n = 8–16). (**B, and right panel of C**) Data are representative of 3–4 pooled mice from a single experiment.

It has been suggested that a possible role for CD103 is the retention of immunoregulatory DCs and T_reg_ cells in the intestinal microenvironment by binding to its ligand E-cadherin. However, it is clear that CD103^−/−^ mice do not develop spontaneous intestinal inflammation and have normal numbers of intestinal DCs [9], [23]. Thus, the exact role of CD103 in intestinal immunity and homeostasis is unclear. Following high-dose *Trichuris* infection in WT mice, we observed an increase in the expression levels of mRNA for CD103 (*Itgae*) in the intestine (**Figure 3A** **, left panel**), but not the mLN (**Figure 3A** **, right panel**). In WT mice, we observed an increase in the frequency of CD11c^+^CD103^+^ DCs in the LP following *Trichuris* infection (**Figure 3B**). Consistent with a previous study [9], we observed equivalent frequencies of CD11c^+^ DCs in the mLN and LP of both WT and CD103^−/−^ mice (**Figure 3C**), suggesting that CD103 is dispensable for DC recruitment and/or retention in the intestine. Thus, the differences observed are unlikely to be due to dysregulated recruitment and retention of DCs in the intestine.

### CD103 is dispensable for T_reg_ cell recruitment and retention in the intestine following *Trichuris* infection

**Figure 4:**
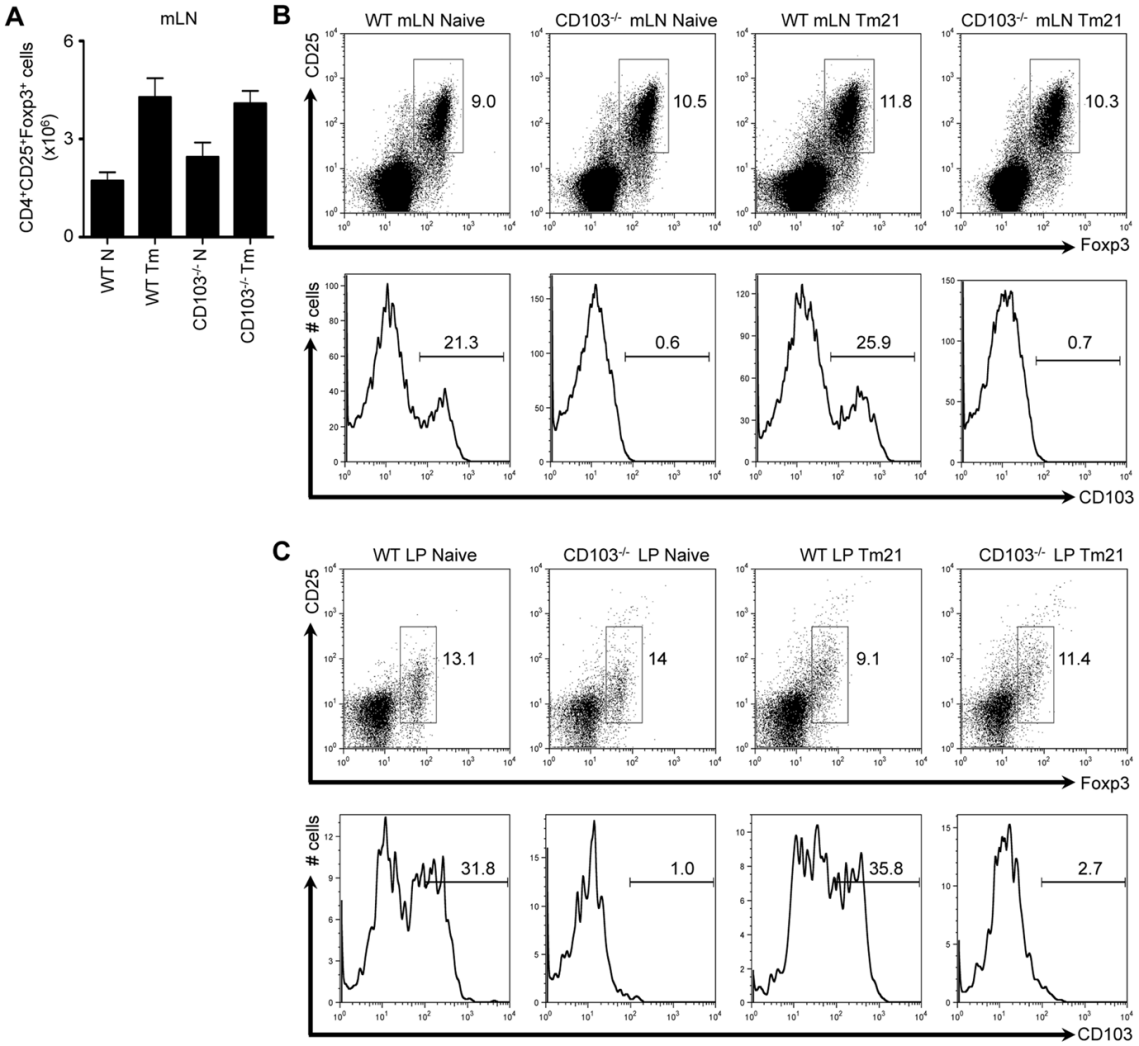
T_reg_ cell recruitment and retention in the intestine following *Trichuris* infection. WT and CD103^−/−^ mice were orally infected with 200 *Trichuris* eggs. (**A**) Number of CD4^+^CD25^+^Foxp3^+^ cells in the mLN of naïve and 21-day infected WT and CD103^−/−^ mice. (**B**) CD25 and Foxp3 expression on CD4^+^ mLN T cells from naïve and 21-day infected WT and CD103^−/−^ mice was assessed by flow cytometry (upper panel). CD103 expression by CD4^+^CD25^+^Foxp3^+^ mLN T cells from 21-day infected WT and CD103^−/−^ mice (lower panel). (**C**) CD25 and Foxp3 expression on CD4^+^ LP T cells from naïve and 21-day infected WT and CD103^−/−^ mice (upper panel). CD103 expression by CD4^+^CD25^+^Foxp3^+^ LP T cells from naïve and 21-day infected WT and CD103^−/−^ mice (lower panel). Data is representative of 2-3 experiments (n = 8–12).

In addition to expression on DCs, CD103 is also expressed on a subset of T_reg_ cells [6], [8], [24]. Thus, the heightened immune response observed following infection with *Trichuris* may be due to a decreased development, recruitment or retention of T_reg_ cells in the intestine. However, the absence of CD103 had no effect on the absolute number of CD4^+^CD25^+^Foxp3^+^ T_reg_ cells in the mLN of naïve or infected mice (**Figure 4A**). Furthermore, the frequencies of CD4^+^CD25^+^Foxp3^+^ T_reg_ cells in the mLN (**Figure 4B**, upper panel) and LP (**Figure 4C**, upper panel) were comparable between WT and CD103^−/−^ mice, both preceding and following infection. Finally, while we observed that approximately 20–30% of T_reg_ cells expressed CD103 in WT mice, as expected, this population was absent in CD103^−/−^ mice (**Figure 4B and 4C**, lower panels). Thus, CD103 is not required for the recruitment and/or retention of T_reg_ cells in the intestine.

## Discussion

We demonstrate that CD103 is largely dispensable for large intestinal immune responses *in vivo*. CD103^−/−^ mice develop slightly heightened T cell responses following acute and chronic *Trichuris* infection. However, lack of CD103 had no effect on parasite expulsion. These surprising results suggest that other mechanisms are in place for the development and maintenance of large intestinal immune responses and homeostasis; possibly including retinoic acid signaling, known to play a role in controlling small intestinal immunity [10], [14], [15], [16], [17], [18]. Alternately, interactions between CCL25 and CCR9, known to specifically target lymphocytes to the small intestine compartment [25], have recently been found to regulate large intestinal immunity in a chemically-induced model of colitis [26], and may play a role in *in vivo* immunity to *Trichuris*.

How CD103 deficiency leads to heightened T cell responses is not clear. One possibility is that in the absence of CD103, tolerogenic, RA-producing DCs are not present. However, we observe equivalent frequencies of CD11c^+^ DCs in the mLN and LP of naïve and infected WT and CD103^−/−^ mice. Consistent with our studies, Annacker *et al.* have reported comparable DC subset composition and distribution in WT and CD103^−/−^ mice [9]. Further, Locksley *et al.* have previously identified a population of functional helper T cells in CD4-deficient mice, indicating that development and function are not necessarily surface marker dependent [27]. Thus, mice may still maintain a functional population of “CD103^+^” immunoregulatory DCs, even in the absence of CD103 expression.

Another possible mechanism is that CD103-dependent signaling is required for inhibition of T cell responses. Consistent with this, it has recently been shown that macrophages and DCs activated in the presence of IL-4 express higher levels of the CD103 ligand E-cadherin *in vitro* and *in vivo* [28]. Furthermore, it has been suggested that E-cadherin-expressing DCs are proinflammatory and promote intestinal inflammation [29]. Supporting a role for CD103 signaling in intestinal homeostasis, Pulendran's group has recently demonstrated that β-catenin in DCs (activated *in vitro* following the disruption of homotypic interactions with E-cadherin [30]) is also involved in the maintenance of intestinal immune homeostasis [31]. Indeed, DC-specific deletion of β-catenin in mice resulted in enhanced inflammation and disease following chemically-induced colitis [31]. Thus, intercellular interactions between CD103 and E-cadherin on IL-4-activated DCs may provide signals–possibly including the induction of RA-synthesizing enzymes such as *Aldh1a2*–that are critical for negatively regulating T cell responses.

Some of the regulatory effects associated with CD103 expression have previously been attributed to the induction and retention of Foxp3^+^ T_reg_ cells [3], [8], [14]. However, CD103^−/−^ mice do not develop spontaneous colitis and we observed normal frequencies of T_reg_ cell populations in the mLN and intestines of naïve and *Trichuris*-infected WT and CD103^−/−^ mice. Furthermore, Powrie's group has previously quantified comparable proportions of CD4^+^CD25^+^ T cells in the spleens of WT and CD103^−/−^ mice [9]. Additionally, expression of CD103 was not essential for T_reg_ cell function within the intestine during inflammation [9]. Thus, it remains unclear what role CD103^+^ intestinal T_reg_ cells play *in vivo*.

In summary, we have demonstrated that CD103 is not required for the development of mucosal T cell immunity in the large intestine during helminth infection. Our results suggest that other not yet identified compensatory mechanisms exist to regulate intestinal immune responses.

## Methods

### Ethics statement

Experiments were approved by the University of British Columbia Animal Care Committee (Protocol number A08-0673) and were in accordance with the Canadian Guidelines for Animal Research.

### Animals, parasites, Ag and infections

C57BL/6 and CD103^−/−^ on a C57BL/6 background were from The Jackson Laboratory. Mice were bred and maintained under specific pathogen-free conditions. Purification of *Trichuris* eggs and antigen (Tm Ag) was performed as described previously [21]. Mice were orally infected with 200 embryonated eggs (acute) for 14 or 21 days, or with 30 embryonated eggs (chronic) for 21 or 32 days.

### Analysis of *Trichuris*-induced immunity

Single cell suspensions from mLN of naïve or *Trichuris-*infected mice were plated at 3–4×10^6^/ml in medium or in the presence of antibodies against CD3 (145-2C11) and CD28 (37.51; 1 µg/ml each; eBioscience) for 72 h. Cytokine production from cell-free supernatants was determined by standard sandwich ELISA using commercially available antibodies (eBioscience). *Trichuris*-specific serum IgG1 and IgG2a levels were determined by ELISA on plates coated with Tm Ag (5 µg/ml). Total protein was isolated from fecal samples, resolved by SDS-PAGE, and immunoblotted using a rabbit anti-mouse RELMβ Ab (PeproTech) [22]. Cecal tissue samples were PFA-fixed and paraffin-embedded. Tissue sections were stained with periodic acid-Schiff (PAS) for visualization of goblet cells. Slides were analyzed on a Zeiss Axioplan2 microscope and images captured using a Qimaging Retiga EX CCD camera and Openlab 4.0.4 software (PerkinElmer).

### Lamina propria cell isolation and flow cytometry

Following sacrifice, the large intestines of either naïve or *Trichuris*-infected mice were removed, opened and washed in PBS. Intestines were incubated at 37°C in PBS containing 2 mM EDTA to remove intestinal epithelial cells, then washed, minced and incubated in 0.5 mg/mL of Collagenase D (Roche) at 37°C for 40 min with rotation. The cells were vortexed and passed through a 70 µm cell strainer (as in [32]). LP cells were washed in cold FACS buffer, resuspended and stained for CD103 (M290), CD11c (N418), CD4 (GK1.5), CD25 (PC61.5) and Foxp3 (FJK16s). Abs were purchased from BD Biosciences or eBioscience. Foxp3 staining was performed using a commercially available kit (eBioscience). Cells were analyzed on a FACSCalibur (BD Biosciences) using CELLQuest software (BD Biosciences).

### RNA isolation and quantitative real-time PCR

RNA was purified from sections of large intestine using mechanical disruption followed by TRIzol according to the manufacturer's instructions. Reverse transcription was used to generate cDNA and qPCR was performed using SYBR green Quantitect primer sets (Qiagen). Reactions were run on an ABI 7900 real-time PCR machine (Applied Biosystems). Samples were normalized against actin and are expressed as fold over naïve.

### Statistics

Results are presented as mean ± SEM of individual animals. Statistical significance was determined by unpaired Student's *t*-test (for parametric data) or Mann-Whitney test (for non-parametric data) using Prism software (GraphPad). Results were considered significant with a *P* value of <0.05.
